# Association of Vitamin K Insufficiency With Cognitive Dysfunction in Community-Dwelling Older Adults

**DOI:** 10.3389/fnut.2021.811831

**Published:** 2022-01-31

**Authors:** Kotaro Azuma, Yosuke Osuka, Narumi Kojima, Hiroyuki Sasai, Hunkyung Kim, Satoshi Inoue

**Affiliations:** ^1^Department of Systems Aging Science and Medicine, Tokyo Metropolitan Institute of Gerontology, Tokyo, Japan; ^2^Research Team for Promoting Independence and Mental Health, Tokyo Metropolitan Institute of Gerontology, Tokyo, Japan

**Keywords:** Vitamin K, undercarboxylated osteocalcin (ucOC), mini-mental state examination (MMSE), mild cognitive impairment (MCI), orientation, calculation, language

## Abstract

Vitamin K is a fat-soluble vitamin shown to be associated with several age-related diseases. Although a small number of epidemiological studies described the relationship between vitamin K status and cognitive impairment, vitamin K status was estimated by relatively special methods in previous reports. Here, we demonstrated the association of the concentration of undercarboxylated osteocalcin (ucOC) in serum, which is a biomarker for vitamin K insufficiency, with cognitive function in a cross-sectional study. A total of 800 community-dwelling older adults (mean age = 75.9) were invited to geriatric health examination, including a Mini-Mental State Examination (MMSE) and a blood test. By using binary logistic regression analysis, the risk of cognitive impairment equivalent or below the mild cognitive impairment level for each tertile of ucOC was examined, with the lowest tertile as the reference. We found a significant association of impaired cognitive function and concentration of ucOC in the highest tertile of ucOC, with the odds ratio of 1.65 (95% CI, 1.06 to 2.59, *P* = 0.028). When the analysis was repeated with each domain of MMSE, the highest tertile of ucOC was associated with impaired orientation, calculation, and language. As far as we know, this is the first report on the significant association of single ucOC measurement and cognitive impairment. Our analysis also suggests that vitamin K insufficiency could be associated with selected categories of cognitive function. Since the single measurement of ucOC in serum is a simple and widely available method for vitamin K evaluation, it could be useful as a biomarker of neurodegenerative diseases affecting the cognitive functions.

## Introduction

Vitamin K is a fat-soluble vitamin originally discovered as an essential factor in blood coagulation. Naturally existing vitamin K compounds are classified into two forms, namely vitamin K1 (phylloquinone) and K2 (menaquinone). Vitamin K1 is abundant in vegetables (1), while vitamin K2 is generated by bacteria and is contained in fermented foods (2, 3). For example, Japanese fermented soybeans (called “natto”) contain high concentrations of vitamin K2 (2). It is also known that the intestinal microbiome is another important source of vitamin K2 production (4).

Functions of vitamin K can be explained by facilitating γ-carboxylation of some proteins, including coagulation factors, osteocalcin (OC), and matrix Gla protein (MGP), which are catalyzed by γ-glutamyl carboxylase (GGCX) (5). To date, other modes of vitamin K actions have been discovered, such as regulation of transcription by activating steroid and xenobiotic receptor (SXR) (6), physical association with 17β-Hydroxysteroid dehydrogenase type 4 (17β-HSD4) (7), and covalent modification of Bcl-2 antagonist killer 1 (Bak) (8).

Of note, several epidemiological studies revealed that vitamin K status is associated with aging-related diseases, including osteoporosis (9) and osteoarthritis (10). Meanwhile, there is a small number of epidemiological studies on the relationship between vitamin K status and cognitive impairment (11–17). In these epidemiological studies, vitamin K status was estimated by food-frequency questionnaires, measurement of dephosphorylated uncarboxylated MGP (dp-ucMGP), or direct measurement of vitamin K concentration by High Performance Liquid Chromatography (HPLC), which will require relatively special methods and/or facilities and may limit translation of these findings into clinical settings.

Osteocalcin (OC) is a protein produced and carboxylated by GGCX in the presence of vitamin K in osteoblastic cells. The concentration of undercarboxylated form of OC (ucOC) in serum is reported to be positively correlated with a fracture risk (18). Furthermore, a high ucOC concentration is used as a biomarker for vitamin K insufficiency for the bone tissue, and as the indicator of vitamin K treatment for osteoporosis in several countries including Japan. The measurement of ucOC is commercially available in relatively low cost compared with other methods, estimating vitamin K sufficiency. Here, we report a cross-sectional study in which the association of ucOC concentration with cognitive function is examined.

## Methods

### Study Design

In 2020, a total of 800 people (mean age = 75.9) were recruited as a follow-up study of the baseline examinations conducted from 2017 to 2019 by mailing the community-dwelling older adults, who were randomly selected from the Basic Resident Register, residing in the Itabashi ward of metropolitan Tokyo. They were invited to a comprehensive geriatric health examination including a Mini Mental State Examination (MMSE) and a blood test. Serum ucOC concentration was measured by BML Inc. (Tokyo, Japan). This study was approved by the institutional ethical committee of Tokyo Metropolitan Institute of Gerontology (approval No.: H18-17, R1-20). All the participants provided written informed consent.

### Statistical Analyses

The levels of serum ucOC were divided into tertiles (T1, T2, and T3). Descriptive statistics were used to compare the characteristics of the patients according to each tertile of ucOC using one-way ANOVA for continuous variables and chi square test for categorical variables. Binary logistic regression analysis was performed to evaluate the association of cognitive impairment (total MMSE score < 27 or 28) (19, 20) with the following variables. Age (continuous), education (binary), sex (binary), hypertension (binary), stroke (binary), heart disease (binary), diabetes (binary), dyslipidemia (binary), osteoporosis (binary), smoking status (binary), body mass index (categorical; < 18.5, 18.5-25, 25–30, and 30+), and ucOC (categorical; T1, T2, T3). Binary logistic regression analysis was repeated to evaluate association of each category of MMSE (orientation, registration, calculation, recall, and language) with the same variables. The cut-off value of each category of MMSE was selected to yield the lowest *P* value in the analysis of the highest tertile of ucOC with the cognitive impairment. The IBM SPSS Statistics version 25 software (IBM Corporation, Armonk, NY, USA) was used for all statistical analyses.

## Results

The characteristics of the study population according to the levels of serum ucOC divided into tertiles (T1, T2, and T3) are summarized in Table 1. The maximum score of MMSE is 30, while the mean MMSE score of the participants in this study was 28.2. In the present study, we focus on cognitive impairment equivalent or below the mild cognitive impairment (MCI) level. Since the cutoff value of MMSE score 27/28 is often employed as the definition of MCI in the study that was evaluating the cognitive function of the Japanese community-dwelling older adults (19), we mainly used this cutoff value. Meanwhile, there is a report proposing the cutoff value of 26/27 is the optimal balance of sensitivity and specificity (20). Among the participants in our study, 204 people (25.5%) had cognitive impairment defined as MMSE score < 28, while 129 people (16.1%) had cognitive impairment defined as MMSE score < 27.

**Table 1:** Characteristics of the study populations according to the levels of ucOC^a^.

**Characteristics**	**ucOC**			***P* value ^b^**	**Total**
	**T1**	**T2**	**T3**		
ucOC (ng/mL)	<2.37	2.37–4.00	> 4.00		
N	267	267	266		800
Age (year)	76.6 ± 4.7	75.0 ± 4.5	76.1 ± 5.3	**<0.001**	75.9 ± 4.9
Female	217 (81.3%)	253 (94.8%)	240 (90.2%)	**<0.001**	710 (88.8%)
Education (year)	12.7 ± 2.3	12.5 ± 2.3	12.9 ± 2.2	0.195	12.7 ± 2.3
Hypertension	111 (41.5%)	108 (40.4%)	122 (45.9%)	0.411	341 (42.6%)
Stroke	3 (1.1%)	6 (2.2%)	11 (4.1%)	0.079	20 (2.5%)
Heart Disease	40 (15.0%)	32 (12.0%)	39 (14.7%)	0.558	111 (13.9%)
Diabetes	50 (18.7%)	24 (9.0%)	29 (10.9%)	**0.002**	103 (12.9%)
Dyslipidemia	136 (50.9%)	116 (43.4%)	128 (48.1%)	0.202	380 (47.5%)
Osteoporosis	109 (40.8%)	55 (20.6%)	55 (20.7%)	**<0.001**	219 (27.4%)
Smokers	78 (29.2%)	54 (20.2%)	59 (22.2%)	**0.038**	191 (23.9%)
BMI (kg/m^2^)	23.1 ± 3.3	23.0 ± 3.1	23.5 ± 3.5	0.175	23.2 ± 3.3
MMSE score	28.1 ± 2.4	28.1 ± 2.4	28.4 ± 1.9	0.254	28.2 ± 2.2

a *Values are means ± SDs for continuous variables or frequency (%) for categorical variables*.

b *P values are for one-way ANOVA (continuous variables) and chi square test (categorical variables)*.

*P < 0.05 are expressed in bold*.

*ucOC, undercarboxylated osteocalcin; BMI, body mass index; and MMSE, Mini-Mental State Examination*.

Next, binary logistic regression analysis was performed to know the association of the cognitive impairment and each characteristic, including age, education, sex, hypertension, stroke, heart disease, diabetes, dyslipidemia, osteoporosis, smoking status, body mass index, and ucOC. Among them, the older age, the longer education years (more than 9 years), and the highest tertile (T3) of ucOC with the lowest tertile (T1) as the reference were significantly associated with cognitive impairment equivalent or below the MCI level defined as MMSE score < 28 (Table 2). The odds ratio (OR) of the highest tertile (T3) of ucOC was 1.65, and its 95% confidence interval (CI) was 1.06 to 2.59 (*P* value, 0.028). When the analysis was performed with another MCI cutoff value “MMSE score < 27,” the results were basically the same; the older age, the longer education years, and the highest tertile of ucOC (T3) were significantly associated with cognitive impairment equivalent or below the MCI level defined as MMSE score < 27. When this cutoff value was employed, the OR of the highest tertile of ucOC (T3) was 1.73, and its 95% CI was 1.01–2.96 (*P* value, 0.045). These results indicated older age, shorter education (<10 years), and vitamin K insufficiency are associated with cognitive impairment.

**Table 2:** Association of characteristics with cognitive impairment.

**Characteristics**	***B***	**OR (95% CI)**	***P* value**
Age	0.153	1.17 (1.12–1.22)	**<0.001**
Sex (women)	0.026	1.03 (0.55–1.90)	0.935
Education (>9 years)	−0.997	0.37 (0.23–0.59)	**<0.001**
Hypertension	0.018	1.02 (0.70–1.48)	0.925
Stroke	−0.120	0.89 (0.30–2.61)	0.827
Heart Disease	−0.258	0.77 (0.46–1.29)	0.323
Diabetes	0.311	1.37 (0.82–2.27	0.230
Dyslipidemia	−0.055	0.95 (0.66–1.37)	0.769
Osteoporosis	0.085	1.09 (0.71–1.66)	0.695
Smokers	0.317	1.73 (0.87–2.16)	0.170
BMI			
<18.5	−0.115	0.89 (0.40–2.00)	0.781
18.5 - 25		Ref.	
25 - 30	0.254	1.29 (0.86–1.95)	0.225
30 <	−0.532	0.59 (0.17–1.99)	0.393
ucOC			
T1		Ref.	
T2	0.014	1.01 (0.64–1.60)	0.953
T3	0.502	1.65 (1.06–2.59)	**0.028**

*B, logistic regression coefficient; BMI, body mass index; CI, confidence interval; MMSE, Mini-Mental State Examination; OR, odds ratio; Ref, reference; ucOC, undercarboxylated osteocalcin*.

*P < 0.05 are expressed in bold*.

The MMSE is composed of five categories, including orientation (maximum, 10 points), registration (maximum, 3 points), calculation (maximum, 5 points), recall (maximum, 3 points), and language (maximum, 9 points). When the analysis was repeated with each category of MMSE, the highest tertile of ucOC was associated with impaired orientation (OR, 7.16; 95% CI, 2.05–27.19; *P* value, 0.002), calculation (OR, 1.52; 95% CI, 1.04–2.24; *P* value, 0.031), and language (OR, 2.44; 95% CI, 1.00–5.94; *P* value,0.049), with adjustment for age, education, sex, hypertension, stroke, heart disease, diabetes, dyslipidemia, osteoporosis, smoking status, and body mass index (Table 3).

**Table 3:** Associations between ucOC and categories of cognitive performances.

	**ucOC**			
	**T1**	**T2**	**T3**
ucOC (ng/mL)	<2.37	2.37–4.00	>4.00
N	267	267	266
MMSE categories			
Orientation (score <9)			
OR (95% CI)	Ref.	0.75 (0.15–3.79)	7.46 (2.05–27.19)
*P* value		0.730	**0.002**
Registration (score <1)			
OR (95% CI)	Ref.	5.38 (0.58–66.23)	6.17 (0.38–22.81)
*P* value		0.184	0.133
Calculation (score <5)			
OR (95% CI)	Ref.	1.36 (0.93–1.99)	1.52 (1.04–2.24)
*P* value		0.108	**0.031**
Recall (score <3)			
OR (95% CI)	Ref.	0.76 (0.50–1.15)	0.78 (0.53–1.22)
*P* value		0.195	0.295
Language (score <8)			
OR (95% CI)	Ref.	0.53 (0.17–1.65)	2.44 (1.00–5.94)
*P* value		0.270	**0.049**

*CI, confidence interval; MMSE, Mini-Mental State Examination; OR, odds ratio; Ref, reference; ucOC, undercarboxylated osteocalcin*.

*P < 0.05 are expressed in bold*.

*The data were adjusted for the following variables: age (continuous), education (binary), sex (binary), hypertension (binary), stroke (binary), heart disease (binary), diabetes (binary), dyslipidemia (binary), osteoporosis (binary), smoking status (binary), and body mass index (categorical; < 18.5, 18.5-25, 25-30, and 30+)*.

## Discussion

In the present study, we demonstrated the association of vitamin K insufficiency as evaluated by serum ucOC with cognitive impairment in the community-dwelling older adult population. As far as we know, this is the first report that focuses on the significant association of single ucOC measurement and cognitive impairment. Furthermore, our analysis suggests that vitamin K insufficiency could be associated with selected categories of cognitive function. Although validation in independent studies will be required, these findings can be a clue to generate hypothesis that vitamin K has important roles in specific areas of brain responsible for several categories of functions.

The finding of the present study was in line with the previous epidemiological studies, showing that lower vitamin K intake is associated with cognitive impairment (11, 12, 14, 16, 17). Among those studies, Kiely and colleagues employed the %ucOC as one of the indicators to evaluate the vitamin K status by measuring both carboxylated OC and ucOC (16). Unfortunately, the relationship between %ucOC and cognitive function was not significant in the study (*P* = 0.06), and the usefulness of ucOC was not discussed in the report. Meanwhile, we showed significant association of vitamin K insufficiency as evaluated by serum ucOC with cognitive impairment, suggesting the possibility that a single measurement of ucOC could be a biomarker for evaluating a vitamin K insufficiency in some epidemiological studies. Since the measurement of ucOC is relatively simple and widely available compared with other methods of vitamin K evaluation, our study would inspire studies on other diseases, as well as cognitive dysfunction, in which vitamin K insufficiency could be involved. In this sense, a protein induced by vitamin K absence-II (PIVKA II), which is an abnormal prothrombin without γ-carboxylation, would be another candidate for efficient evaluation of vitamin K status in the epidemiological studies (21).

It remains unknown whether the undercarboxylated form of osteocalcin itself affects the cognitive function. It is also possible to consider that a high concentration of ucOC merely reflected a low vitamin K intake, and that other substrates of GGCX are responsible for the mechanism. Previous studies with an animal model suggested Gas6 and Protein S, both of which are substrates of GGCX and have neuroprotective effect. The Gas6 was reported to prevent apoptosis of neuronal cells (22), oligodendrocyte loss, and microglial activation (23). Also, Gas6 is reported to prevent amyloid beta protein-induced apoptosis of cortical neuron (24), suggesting its protective role against Alzheimer's disease. Protein S is shown to be associated with neuronal protection against ischemic injury (25), suggesting its beneficial role for cognitive impairment due to vascular dementia. It is also possible that other γ-carboxylated proteins including unidentified ones expressed within the brain or expressed outside the brain, which enable to pass through a blood brain barrier, would be important for brain protective effect. In human, a single nucleotide polymorphism (SNP) of *GGCX* gene is shown to be associated with risk susceptibility of stroke when combined with SNP of NAD(P)H:quinone oxidoreductase (*NQO1*) gene, which encodes a protein protective against oxidative stress (26). The SNP of GGCX is responsible for the difference of an amino acid (Gln325Arg), while the protective SNP against stroke encodes GGCX with higher activity (325Gln) (26, 27).

Since vitamin K has multiple modes of action (5), other mechanisms independent of GGCX could be involved in its effects on cognitive impairment. A nuclear receptor, SXR, for which vitamin K functions as a ligand (6), is shown to have anti-inflammatory roles (28). Among vitamin K compounds, the vitamin K2 form is known to activate SXR (6). In a study analyzing the postmortem brains of centenarians, MK-4 form of vitamin K2 was detected in human brains (17). Meanwhile, expression of SXR was reported in the capillaries in rat brains (29), which supports the existence of SXR-dependent vitamin K function in the brain. The relationship of inflammation and cognitive impairment is proposed including the indirect immune pathways from the gut microbiome and systemic circulation (30). Considering the SXR knockout mice display intestinal inflammation (28), SXR might mediate beneficial functions of vitamin K on cognitive functions by suppressing inflammatory response of the intestine. Vitamin K in the body is partially derived from intestinal microbiome (4), and the relationship of several forms of microbiome-derived vitamin K, and the cognitive function emerged as a new study topic (11).

It is an intriguing question to know the blood concentration of vitamin K is correlated with vitamin K concentration in brain. Although we can assume function of GGCX or SXR outside the brain could affect brain function *via* circulating γ-carboxylated proteins or inflammatory mediators, some hypothesis may depend on the GGCX or SXR functions within the specific area of the brain. In the study analyzing postmortem brains of centenarians, the concentration of vitamin K in the blood did not correlate with that in the brain (17). Meanwhile, in other reports, measurement of vitamin K concentration in the brain decreased with the storage time of the brain (31), which may affect the study using centenarians.

In summary, the epidemiological studies, including the present study, will promote future studies of vitamin K on a higher brain function including basic studies and clinical intervention studies.

## Data Availability Statement

The raw data supporting the conclusions of this article will be made available by the authors, without undue reservation.

## Ethics Statement

The studies involving human participants were reviewed and approved by Institutional Ethical Committee of Tokyo Metropolitan Institute of Gerontology (approval number: H18-17, R1-20). The patients/participants provided their written informed consent to participate in this study.

## Author Contributions

KA, YO, NK, and HS acquired the data. KA conducted the data analyses. All authors contributed to the interpretation of data, drafted the manuscript, approved the final version for publication, and conceived and designed the study.

## Funding

This study was funded by TMIG institutional project funding (SASM). The funder did not have any role in the study design, analyses, interpretation of the results, decision to publish, or in the preparation or approval of the manuscript.

## Conflict of Interest

The authors declare that the research was conducted in the absence of any commercial or financial relationships that could be construed as a potential conflict of interest.

## Publisher's Note

All claims expressed in this article are solely those of the authors and do not necessarily represent those of their affiliated organizations, or those of the publisher, the editors and the reviewers. Any product that may be evaluated in this article, or claim that may be made by its manufacturer, is not guaranteed or endorsed by the publisher.
